# Ocular Surface Inflammatory Disorders (OSID): A Collective of Systemic Etiologies Which Cause or Amplify Dry Eye Syndrome

**DOI:** 10.3389/fmed.2022.949202

**Published:** 2022-07-06

**Authors:** Matias Soifer, Nadim S. Azar, Hazem M. Mousa, Victor L. Perez

**Affiliations:** ^1^Foster Center for Ocular Immunology, Duke Eye Institute, Durham, NC, United States; ^2^Department of Ophthalmology, Duke University Medical Center, Durham, NC, United States

**Keywords:** dry eye, inflammation, conjunctiva, lacrimal gland, meibomian gland, ocular surface inflammation, inflammatory dry eye

## Abstract

The ocular surface inflammatory disorders (OSID) are caused by systemic disorders that conduct a persistent inflammatory reaction in the ocular adnexal connective tissues, such as the conjunctiva, lacrimal gland (LG) and meibomian glands (MGs), which cause an inflammatory dry eye. The etiologies of OSID are a subset of systemic pathologies such as graft versus host disease, Sjögren’s syndrome, allergies, cicatrizing conjunctivitis, and more. These cause a purely inflammatory dry eye syndrome as a consequence of the persistent surrounding inflammation in the adnexal tissues, which is distinct from the age-related dry eye disease. A limitation toward management of these conditions is the lack of available biomarkers that can detect presence of inflammation and quantify damage on the conjunctiva and LG, even though these are considered to be drivers of the inflammatory milieu. The OSID and dry eye syndrome are caused by different immune cells which are not exclusively limited to T cell lymphocytes, but rather derive from an orchestrated multicellular immunologic response. Recognition of this syndrome is crucial to direct research in a direction that clarifies the potential role of inflammation and its associated immune phenotype on the conjunctiva and adnexal ocular tissues in OSID and dry eye syndrome. On this paper, we review the basic and clinical research evidence for the existence of OSID with focus on the different immune cells involved, the target tissues and potential consequences and OSIDs diagnostic and therapeutic implications.

## Introduction

Dry eye disease (DED) is a multifactorial ocular surface disorder which causes signs and symptoms on a high global estimated prevalence that ranges from 5 to 50% (1).

This is a relevant and complex pathology which continues to be misclassified and mistreated despite facing a growing prevalence and producing a substantial societal cost. Commonly, diagnostic tests for DED are focused on corneal damage and tear film biomarkers, which detect the end result on the cornea and tear film, meanwhile the conjunctiva, lacrimal gland (LG), meibomian glands (MGs), and ocular adnexal tisues are often underappreciated. Importantly, these tissues are in different degrees known targets of evident inflammation in systemic inflammatory disorders, such as ocular allergies, rosacea, Sjögren’s syndrome (SS), graft versus host disease (GVHD), different cicatrizing conjunctivitis syndromes and more. These share a key element in common, which is the infiltration of inflammatory cells in the ocular local connective tissues, that results in Ocular Surface Inflammatory Disorders (OSID), which cause an inflamed and dry ocular surface environment. The OSID are instigated by a subset of systemic pathologies that conduct inflammation in local ocular connective tissues and causes a purely inflammatory DED. We will review the OSID with experimental and *in vivo* evidence for their existence, immunological basis, target tissues and implications. Finally, we will dive into current pharmacologic therapies for dry eye and OSIDs and future directions.

### Methodology and Goal of the Review

The goal of this review is to present a narrative perspective on OSID assimilating studies published in the literature integrating evidence for the role of adnexal inflammation on the inflammatory dry eye circuit. The review is based on pertinent publications from 1990 to 2021 using the terms “ocular surface inflammation” “Dry eye”; “Conjunctiva”; “Lacrimal gland”; Meibomian gland,” “ocular graft versus host disease,” “Sjögren’s,” “Stevens Johnson’s,” “allergy,” “pemphigoid.” These were retrieved by a selective PubMed search and on the authors’ own clinical and scientific experience.

### Ocular Surface Inflammatory Disorders

**FIGURE 1:**
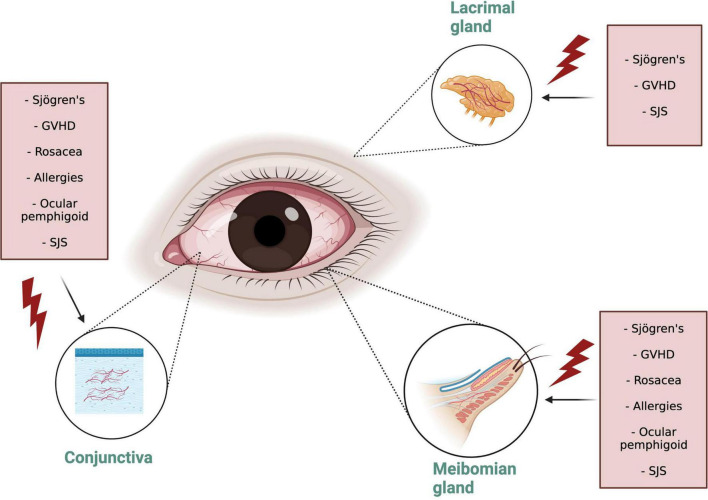
Ocular Surface Inflammatory Disorders (OSID) target organs. Note that the Diverse OSID impact in different degrees all the adnexal connective tissue structures. OSID, ocular surface inflammatory disorders; GVHD, graft versus host disease; SJS, Stevens Johnsons syndrome.

Dry eye disease is an “umbrella” term that encompasses various disorders of the ocular surface, such as evaporative dry eye or aqueous deficient dry eye. Alternatively, DED can be classified according to the symptoms as neurotrophic or neuropathic pain. Similarly, DED can be classified as being caused by a systemic inflammatory disorder or not. The OSID occur when there is persistent presence of adnexal connective tissue inflammation as a result of a systemic immunologic disorder, which precipitate a dry eye syndrome either by causing it or amplifying it. They are commonly the result of autoimmune disorders that involve an influx of inflammatory cells to the conjunctiva, MGs, lids, and the LG (Table 1). These disorders conduct inflammation even though they do not represent the common condition of keratoconjunctivitis sicca or “age related” DED (2). In this way, the OSID are a pure form of inflammatory dry eye that arises from the systemic inflammatory influx to the ocular surface and adnexal tissues. As an example, in ocular GVHD, the DED appears as a result of the attack of the donor T cells in the hosts ocular surface after the hematopoietic stem cell transplant. The DED is a consequence, not a cause, of the inflammatory insult. The OSID collective pathological sign is cellular inflammation that chronically infiltrates the subconjunctival ocular surface tissues, MGs, and LG, further perpetuating the autoinflammatory insult to the ocular surface, causing loss of homeostasis that results in signs and symptoms of DED (Figure 1). The findings include: (1) Persistent conjunctival leukocyte infiltration, leading to conjunctival vasodilation and trasudation (3); (2) Cellular infiltration around meibomian glands with release of myeloperoxidase (4), leading to meibomian gland disorders (5); (3) Cellular infiltration into the LG acini, which leads to gland atrophy and decreased tear production (6); (4) Infiltration into the conjunctiva subepithelial layer leading to direct inflammatory injury of the conjunctival epithelium (7); (5) Low-grade inflammatory infiltration into the cornea leading to corneal nerve damage and/or injury to the corneal epithelium (8–10). Nonetheless, the confirmatory diagnosis of these disorders includes a conjunctival biopsy which is seldomly performed, since these illnesses are diagnosed clinically. Importantly, although the role of the tear film and corneal epithelial cells have been extensively described, that of LG and conjunctiva remains far less explored clinically, even though these are considered to be drivers of the inflammatory milieu.

**TABLE 1:** Ocular surface inflammatory disorders (OSID) and “reported prevalence” of dry eye disease.

Etiologies	Immunologic main mechanism	Prevalence of DED
Graft Versus Host Disease	Donor T-lymphocytes, specifically CD8 lymphocytes, attack ocular adnexal tissues	50–60% (24)
Primary Sjögren syndrome	Lymphocytic infiltration in lacrimal gland	88.1–94% (58, 59)
Stevens Johnsons Syndrome	Type 3 hypersensitivity on adnexal tissues	27–59% (60)
Ocular Allergic Disorders	IgE hypersensitivity response and/or cell-mediated responses on conjunctiva	28.4% (59)
Rosacea	Cellular pattern recognition receptors and dysregulated inflammatory mediators on conjunctiva and meibomian glands	17.6% (59)
Ocular Cicatricial Pemphigoid	Immunoglobulin or complement component deposition at the epithelial basement membrane zone in the conjunctiva	68–77.3% (61)
Rheumatoid Arthritis	Lymphocytic infiltration of lacrimal glands (secondary Sjögren’s syndrome)	6–53% (62)
systemic lupus erythematosus	Tissue-binding autoantibodies and immune complexes. mononuclear cell infiltration of both the major and accessory lacrimal glands.	39.5% (63)
Mixed-connective tissue disease	Tissue-binding autoantibodies and immune complexes	14.5–56% (64)

### The Link Between Ocular Surface Inflammatory Disorders and Dry Eye Disease: Inflammation

One key factor that has been repeatedly recognized in DED is the role of inflammation (11), which is thought to act in a vicious cycle fashion as the disruption of the wet ocular surface environment promotes influx of inflammatory cells, that in turn injuries the epithelium and adnexal structures, promoting the persistence of an unstable ocular surface. Even though the presence of inflammation in the DED cycle is undeniable, the nature of this inflammation is unresolved and controversial. One of the pivotal elements responsible for the inflammatory casqued are the T Cell Lymphocytes which after being recruited and activated in the ocular surface release cytokines that perpetuate the existence of an inflamed ocular surface microenvironment. Evidence for this comes from mouse models of dry eye, on which CD4 T cells were transferred to T-cell–deficient mice, with a consequent inflammation in the LG, cornea, and conjunctiva, resulting in decreased tear production and conjunctival goblet cell loss (12). Note that the influx of inflammatory cells was not only observed on the cornea, but similarly on the adnexal connective tissues. Likewise, in experimental dry eye models treated with topical cyclosporine (an immunomodulatory agent of T cell lymphocytes) a successful inhibition of dry eye mediated conjunctival epithelial apoptosis was observed (13). The evidence for inflammation is also noticeable through tear film proinflammatory cytokine detection which revealed an increased detection of Interleukin (IL)-1, IL-6, and IL-8 in dry eye patients (14, 15). Interestingly in diverse OSID, similar mechanisms of disease and results have been demonstrated, such as presence of these cytokines in tear film of ocular GVHD patients (16), SJS (17) and ocular cicatricial pemphigoid (OCP) (18) compared to normal controls. Data for positive presence of inflammation on these conditions is likewise observable in impression cytology of bulbar and tarsal conjunctiva (19) and through *in vivo* confocal imaging of the ocular surface (20). Importantly, we and others have consistently reported dry eye disease signs and symptoms on patients suffering from OSIDs (21–23).

However, the scope of ocular surface inflammation in DED and OSID is not only dependent of T effector lymphocytes, but is rather an orchestrated response that combines the adaptive and innate immunity as well, namely through macrophages (24) and neutrophiles (25–28). These immune elements are responsible for the initial or acute phase of DED driven *via* a non-specific innate immune response which is typically followed by an adaptive immune response. Most patients will present episodic rebounds of inflammation which present with an exacerbation of signs and symptoms of ocular surface instability commonly caused by diverse factors (29) including exacerbation of systemic inflammatory disorders (3). Essentially, the hyperosmolar stress created has a direct pro-inflammatory effect on the ocular surface epithelial cells which respond by activating mitogen-activated protein kinases (MAPKs) (30), stimulating secretion of pro-inflammatory chemokines and cytokines (31), in addition to matrix metalloproteinases (32, 33). These contribute to the inflammatory cascade by cleaving pro-cytokines and establishing chemokine gradients attracting more inflammatory cells and increasing the hyperimmune drive.

It is thought that the antigen presenting cells (APCs) phagocyte and later present to naïve T cells autoantigens, which are theorized to be exposed by the desiccating stress or altered cell differentiation. While antigen presentation is often perceived to be their main role, all these mononuclear phagocytes also secrete proinflammatory cytokines (TNF-α, IL-6, and IL-12) and chemokines when activated. Recently, plasmacytoid dendritic cells have also been recognized as a driver of inflammation (34). The components of the innate cell response described play a key role in the initiation and propagation of the DED and this might explain why most FDA approved anti-inflammatory therapies for DE (which target exclusively T cell inhibition) have shown only partial and/or moderate improvement in signs and/or symptoms of DED in clinical trials: These drugs do not possess a mechanism of action to completely suppress all the immune cell players in the cycle of inflammation. A limitation of DED clinical trials is a lack of differentiation of the included study population in OSID versus “non-OSID” dry eye since the ocular surface of the latter is not under the same persistent adnexal inflammatory influx as the OSID. Recognizing the syndrome allows for better categorization of study populations which will impact trial results and ultimately patient management guidelines.

### Evidence of Ocular Surface Inflammatory Disorders Mechanisms in Experimental Dry Eye Models

While *in vivo* human evidence for the conduction of OSID into DED is scarce, animal models have provided useful data for its mechanisms as these emulate the intrinsic mechanisms of OSID to produce a DED. We have learned that APCs play a decisive role in linking the innate immune with the adaptive immune response *via* chronic CD4 + T cell-driven response (35). This was validated through animal models in which diminished APC populations inhibited the generation of autoreactive CD4 + T cells and blocked disease progression (36). When active, the T-cell response results in a plethora of ubiquitous inflammatory components that carry out DED progression (12). Such components consist of a variety of inflammatory cytokines mainly Th1 and Th17 related, as well as other mediators, which result in an immune-mediated damage of the ocular surface (37). In addition to the aggravated abundance of pro-inflammatory agents, a dysfunctional immune-regulatory unit is equally complicit in the pathogenesis of DED and OSIDs. Reduction in either quantity or quality of T-regulatory cells (T-regs) in murine models results in exacerbated disease, highlighting their key role in regulating inflammation of the ocular surface (38). Th-17 cells and associated cytokines have been consistently implicated in a variety of autoimmune ocular surface diseases such as SS, GVHD, and allergic eye disease (38–41). Moreover, Recent studies have suggested that pro-inflammatory components in the tears plays a key role in the pathogenesis of dry eye disease as was seen in SS (42) GVHD (43), and allergic eye disease (26). Animal model limitations are their translational obstacles, since mice have different anatomical components (nictitating membrane and a harderian gland) which may differ from human tear film dynamics. Also, these have one specific manipulated disorder (gene knockout), whereas humans tend to have a myriad of comorbidities. Plus, the conditions in which mice are maintained are “sterile,” as compared to the human daily environmental challenges. Lastly, there is an inability to detect and measure symptoms of mice, as these represent a big burden on patients. As such, the delicate balance between pro-inflammatory mediators, innate and adaptive immunity cells has been revealing that OSIDs, *via* different inciting events, cause an inflamed ocular surface which culminates in dry eye.

### Damage to the Target Organs in Ocular Surface Inflammatory Disorders and Potential Implications

**FIGURE 2:**
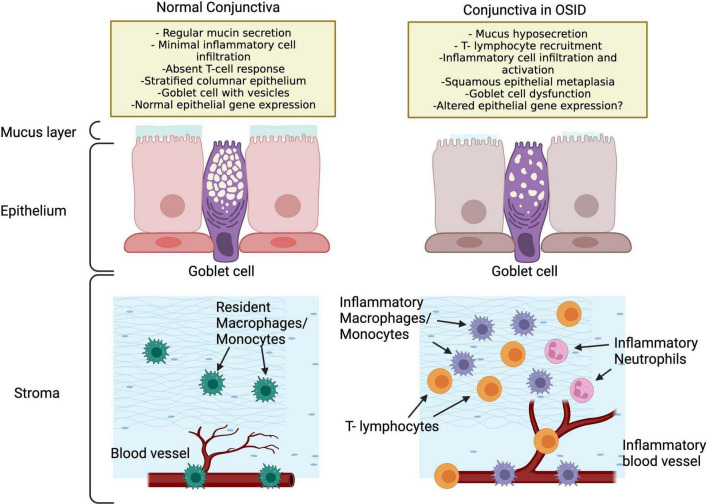
Potential conjunctival changes in OSID that lead to ocular dryness and increased inflammation. Note that the OSID impact the conjunctiva in many ways that are not fully appreciated.

The conjunctiva is a mucous membrane that provides coverage for the ocular surface, composed of a stratified epithelium and an underlying loose stroma, which is mainly composed of Collagen IV. The conjunctival epithelial cells produces water, through aquaporins (44), mucins (45) and proteins such as lubricin (46). On the outermost epithelial layer, mucin is secreted from intraepithelial vesicles, which forms a glycocalyx that confers wettability to the hydrophobic epithelium surface anchoring the soluble mucin to the conjunctival surface. These wide range of functions makes the conjunctiva a key element in the maintenance of ocular surface homeostasis and, at the same time, quite reactive to small environmental changes and even prone to alterations. In OSID, the persistent presence of leukocytes in the conjunctiva alters its functions in ways that are not fully appreciated (Figure 2).

In conjunctival biopsies with immunohistochemistry of SS patients, a lymphocytic infiltration has been documented with CD4 + T prevalence, and in a lower quantity of CD8 + T cells and B cells (47). Additionally, impression cytology specimens from SS eyes, as compared to controls and other conditions, present more squamous metaplasia, goblet cell loss and inflammatory cells intercalated with epithelial cells (48). Can the persistent conjunctival inflammation play a role in modifying gene expression of epithelial cells? A recent study comparing the conjunctival gene expression of SS patients with controls resulted in the discovery of 53 differentially expressed genes between both groups, indicating that SS patients, and potentially the OSIDs, exhibit a phenotype of immune activation which contributes to dysregulation of the conjunctiva and ocular surface (7). Interestingly, SS patients have also been reported to exhibit greater number of lymphocytes in the tarsal conjunctiva than controls (49), which supports that OSIDs have an impact not only the classically defined targets (as the LG in in SS), but likewise in the conjunctival epithelium (48) and MG (49) (Figure 1).

In different OSIDs, similar observations have been made: In the conjunctiva of patients with OCP, high expression of IL-8 and its receptor were noted *via* impression cytology (50). In GVHD, epithelial HLA-DR expression and CD8-positive lymphocytes were more frequently observed than in controls. Additionally, in patients with vernal keratoconjunctivitis a decrease in goblet cell density has been detected (51), which, when treated with cyclosporine, exhibited a significant increase (52). This advocates that OSIDs damage the conjunctiva in many ways, but likewise that decreasing the inflammatory state aids in conjunctival functionality, which further corrects the ocular dryness.

Concerning the tarsal conjunctiva and MGD, it is being more understood that inflammation plays a key role in its dysfunction. In GVHD, notorious damage to the MG occurs (22) and *via* IVCM, infiltration of inflammatory cells in the MG has been noted, supporting that the inflammation is responsible for the extensive damage to these glands (53). Likewise, in a chronic inflammatory model of allergic eye disease in mice, it was shown that neutrophils promoted meibomian glands obstruction (26). This was shown as well, *in vivo* on human patients, on whom analysis of leukocytes in tears of MGD showed an increase in PMN numbers compared to healthy subjects (26). Collectively, these findings argument a role for OSID in conducting immune mediated MGD.

The LG is another known target of OSID in preclinical models, however *in vivo* data is hard to compile, mainly because there are not currently available devices for LG observation on a cellular level. In SS, LG biopsies showed a progressive lymphocytic infiltration and an increase of pro-inflammatory cytokines with consequent acinar atrophy, tissue fibrosis, and interlobular inflammation (54, 55). In mice models of GVHD immunohistochemistry sections of the LG revealed periductal fibrosis and dense infiltrates of macrophages and lymphocytes (56). These further indicates that in OSIDs potentially all the structures of the ocular adnexa are involved in the inflammatory drive, which synergistically leads to a dry and inflamed ocular surface.

### Current Anti-inflammatory Therapies for Dry Eye: Acute Episodes

Acute management classically derives from topical corticosteroids which can down-regulate both innate and adaptive immune response pathways and inhibit signaling pathways and transcription of relevant cytokine and chemokines within hours. In mouse models of DED, corticosteroids suppress IL-1, IL-6, MMP-9 as well as activation of MAPKs stress-signaling (57). However, known side effects limit their long-term use. Recently, Eysuvis (loteprednol etabonate 0.25%, Kala pharmaceuticals) was granted FDA approval for the short-term treatment of the signs and symptoms of dry eye disease for the first time.

### Current Anti-inflammatory Therapies for Dry Eye: Chronic Disease

Because of the adverse effects provoked by steroids it is of extreme importance to possess (as in other rheumatologic inflammatory disorders) steroid sparing therapies for chronic inflammatory dry eye management. For this, only three medications are approved by the FDA, and these are Restasis (Cyclosporine 0.05%), Xiidra (lifitegrast 5%) and Cequa (Cyclosporine 0.09%). Restasis (Allergan) inhibits the production of cytokines involved in the regulation of T-cell activation. It received FDA approval for dry eye presumed to be triggered by inflammation by showing increased tear production, as compared to controls. Cequa (Sun pharmaceutical industries), was approved for the same indication as Restasis. Xiidra (Novartis), is a lymphocyte function-associated antigen 1 (LFA-1) antagonist, which received approval for treatment dry eye signs and symptoms, after demonstrating better outcomes of inferior corneal staining and symptoms measured than placebo. These three drugs act by inhibiting the adaptive immunity, through exclusive T cell inhibition but may not act against the other cell lineages that have been discussed previously leaving a potential gap in their mechanism of action toward halting inflammation in DED. Additionally, these have only shown statistically significant differences in regard to one or two components of the broad manifestations that OSID and DED can cause because these drugs were approved on the basis of tear film and/or corneal parameter changes, without evaluating the impact on the adnexal elements (LG, conjunctiva, and meibomian glands) discussed. Hence, current anti-inflammatory therapies do not necessarily represent a cure, but rather are being approved on their capacity to attenuate the final insult of OSID and DED on the cornea or the tear film. This paradigm disregards the progression of disease in the lacrimal gland, conjunctiva, and adnexal tissues, which are key targets in the origin, propagation, and termination of OSID.

## Conclusion

Ocular surface inflammatory disorders (OSID) are caused by a collective of systemic disorders that produce a spectrum of inflammatory reactions in the ocular adnexal connective tissues causing an inflamed ocular surface. The resulting dry eye disease is a consequence, not a cause, of the surrounding inflammation. A limitation toward management of these conditions is the lack of available biomarkers that can detect presence of inflammation and quantify the damage to the conjunctiva and LG.

Recognition of the OSID entity as a sub classification of DED is crucial to direct research in a direction that clarifies the potential role of inflammation and its associated immune phenotype on the conjunctiva and adnexal ocular tissues as well as the ocular surface and cornea both in OSID, as in non-OSID DED. This will lead to an understanding of how to categorize these populations in clinical trials, what anti-inflammatory therapies must be diligently selected to dampen the inflammatory insult, and what features should be selected on a drug to successfully penetrate a deeply inflamed conjunctival tissue.

## Data Availability Statement

The original contributions presented in this study are included in the article/supplementary material, further inquiries can be directed to the corresponding author.

## Author Contributions

MS and VP: conception or design of the work. MS, HM, and NA: data collection and data analysis and interpretation. MS: drafting the article. NA: figures edition. VP: final approval of the version to be published. All authors contributed to the article and approved the submitted version.

## Conflict of Interest

VP was employed by Alcon, Evolve by Nature, Pelican Pharma; Asclepix, Azura, Dompe, Dompe, Kala, Kiora, Novartis, Oyster Point, Sight Sciences, Sun Pharma. The remaining authors declare that the research was conducted in the absence of any commercial or financial relationships that could be construed as a potential conflict of interest.

## Publisher’s Note

All claims expressed in this article are solely those of the authors and do not necessarily represent those of their affiliated organizations, or those of the publisher, the editors and the reviewers. Any product that may be evaluated in this article, or claim that may be made by its manufacturer, is not guaranteed or endorsed by the publisher.
